# Preclinical and clinical phase I studies of a new recombinant Filgrastim (BK0023) in comparison with Neupogen^®^

**DOI:** 10.1186/2050-6511-15-7

**Published:** 2014-02-21

**Authors:** Davide Crobu, Gaia Spinetti, Rodolfo Schrepfer, Giancarlo Tonon, Gloria Saccani Jotti, Pierluigi Onali, Simona Dedoni, Gaetano Orsini, Andrea Di Stefano

**Affiliations:** 1 Bio-Ker S.r.l.-Multimedica Group, Sardinia Scientific and Technological Park, Building 3, I-09010 Pula Cagliari, Italy; 2 IRCCS MultiMedica, Laboratory of Diabetological Research, Milan, Italy; 3 Department of Biomedical Sciences, Laboratory of Cellular and Molecular Pharmacology, University of Cagliari, 09042 Monserrato Cagliari, Italy; 4 CROSS Research S.A, Phase I Unit, via F. A. Giorgioli 14, CH-6864 Arzo, Switzerland; 5 Department of Biomedical, Biotechnological and Traslational Science (S.Bi.Bi.T.), University of Parma, via Volturno 39, 43121 Parma, Italy

**Keywords:** r-Met-G-CSF, BK0023, Biosimilar Filgrastim, Comparability

## Abstract

**Background:**

Filgrastim or methionyl-granulocyte colony-stimulating factor (Met-G-CSF), is a recombinant therapeutic protein widely used to treat severe neutropenia caused by myelosuppressive drugs in patients with nonmyeloid malignancies. In addition to its role in the regulation of granulopoiesis, treatment with G-CSF is considered the standard approach to mobilize CD34 positive (CD34^+^) mononuclear cells for reconstituting hemopoietic ability for bone marrow transplantation. An intended biosimilar filgrastim (coded BK0023) was produced in GMP conditions by *E.coli* fermentation according to an original recombinant process and showed physico-chemical properties and purity profile similar to Neupogen^®^, a commercial preparation of filgrastim. The aim of the present study was to demonstrate the comparability of BK0023 to Neupogen^®^ in terms of both *in vitro* biological activities and *in vivo* toxicology, pharmacokinetics and pharmacodynamics.

**Methods:**

Cell proliferation and radioligand binding assays were conducted in NFS-60 cells to compare the biological activity and functional interaction with the G-CSF receptor *in vitro,* while preclinical *in vivo* studies, including pharmacokinetics and pharmacodynamics after repeated dose were performed in normal and neutropenic rats. A phase I study was carried out in healthy male volunteers treated by multiple-dose subcutaneous administration of BK0023 and Neupogen^®^ to evaluate their pharmacodynamic effects as well as their pharmacokinetic and safety profile and to demonstrate their pharmacodynamic equivalence and pharmacokinetic bioequivalence.

**Results:**

The results reported in this work demonstrate that BK0023 is comparable in terms of biological activity, efficacy and safety to Neupogen^®^.

**Conclusions:**

BK0023 has the same pharmacokinetic profile, efficacy and safety as the reference commercial filgrastim Neupogen^®^ and therefore could be further developed to become a convenient option to treat neutropenia in oncological patients.

**Trial registration:**

Trial registration number (TRN): NCT01933971. Date of registration: Sept 6th 2013.

## Background

Human granulocyte-colony stimulating factor (G-CSF) is a haematopoietic glycoprotein produced by stromal cells, macrophages, endothelial cells, fibroblasts and monocytes which binds with high affinity to the G-CSF receptor expressed on neutrophilic precursor cells in the bone-marrow, inducing them to proliferate and differentiate into infection-fighting neutrophils without significant haemopoietic effects on other lineages of blood cells.

The use of recombinant G-CSF preparations is a well established treatment for accelerating bone marrow recovery, for preventing the onset of severe myelosuppression and its correlated complications and for reducing febrile neutropenia in patients with non-myeloid malignancies under radio- or chemotherapies [1,2]. Moreover, G-CSF is clinically used as bone marrow mobilizer to increase the number of circulating CD34^+^ progenitor cells [3] employed for hematopoietic cell reconstitution. Besides, since recent preclinical evidences showed a pro-angiogenic potential of CD34^+^ cells, the use of these cells for regenerative medicine of ischemic diseases is currently under investigation in clinical trials [4]. Other studies suggest that G-CSF is worth investigating as an interesting therapeutical option in cardiovascular field for the prevention of restenosis after percutaneous coronary intervention [5] as well as for its beneficial effect in stroke recovery due to the enhancement of neurogenesis and the stimulation of blood vessel formation [6].

In response to the need of a highly purified protein for medical applications, both glycosylated and non-glycosylated forms of human G-CSF have been manufactured by recombinant DNA technology. Glycosylation appears to increase resistance of G-CSF to proteolysis, but it is not required for receptor binding or biological activity of the protein. In fact, recombinant glycosylated and non-glycosylated G-CSF variants exhibit the same pharmacokinetics and pharmacodynamic profiles [7].

Recombinant Met-G-CSF or filgrastim is a single-chain protein of 175 aminoacids and two disulfide bonds differing from the native G-CSF in that it is not O-glycosylated on threonine 134 and bears an additional N-terminal methionine required for expression in *E. coli*. An original method to produce a biosimilar filgrastim, called BK0023, by high yield bacterial expression of a fusion protein in the form of inclusion bodies was developed. After *in vitro* refolding, the fusion protein was enzymatically cleaved to give functionally active r-Met-G-CSF which was purified and characterized. Finally, we conducted *in vitro* and *in vivo* preclinical studies as well as a clinical phase I study in healthy volunteers to compare the biological, pharmacodynamic, pharmacokinetic and safety profile of BK0023 and Neupogen^®^.

The results demonstrated the pharmacodynamic equivalence and the pharmacokinetic bioequivalence of BK0023, a recombinant filgrastim produced according to an original process, towards Neupogen^®^, a proprietary filgrastim approved for clinical use, and support the claim for further assessments of BK0023 as biosimilar drug that, once approved for marketing, could potentially be a useful and cost-effective treatment option for patients requiring G-CSF.

## Methods

### Cell culture

Murine myeloblastic NFS-60 cells were purchased from Cell Line Service (Eppelheim, Germany). RPMI 1640 medium, fetal bovine serum (FBS), fetal calf serum (FCS), penicillin and streptomycin were provided by Gibco Life Technologies (Monza, Italy). Mouse interleukine 3 (IL-3) was from Sigma-Aldrich (St. Louis, MO, USA) while WST-1 reagent was from Roche (Milan, Italy). Materials for gel electrophoresis were provided by Bio-Rad Laboratories Inc. (Hercules, CA, USA). If not otherwise reported, all other reagents and chemicals were of analytical grade and provided by Sigma Aldrich-Fluka, Merck or Carlo Erba (Milan, Italy).

### Test products

Recombinant Met-G-CSF (BK0023) was produced using a Bioker’s manufacturing technology at Eurogenetec S.A., Belgium, in GMP conditions by high biomass *E.coli* fermentation according to a previously described protein fusion technology starting from a gene coding for filgrastim bearing an N-terminal enhancer peptidic tag [8]. Briefly, LacZ_8_-PNP_20_-Kex-1-Met-G-CSF hybrid fusion protein (where LacZ8 represents the first 8 aminoacids of the LacZ protein fragment [UniProt/SwissProt accession Q37953], PNP_20_ is a sequence coding for the first twenty amino acids of *E.coli* purine nucleoside phosphorylase [9] and the N-terminal methionine residue of filgrastim is immediately preceded by a sequence coding for the short flexible peptide -Glu-Ser-Ser-Met-Ser-Gly-Leu-Phe-Lys-Arg- ending with a Lys-Arg basic dipeptide which *in vitro* is specifically cleaved at C-terminal site of arginine by the endoprotease ss-Kex-1-C_611_ to leave the mature sequence of filgrastim) was expressed in *E.coli* as cytoplasmic inclusion bodies. After isolation of inclusion bodies, the fusion protein was dissolved in 7 M guanidine, renaturated by dilution, dialyzed and cleaved by treatment with the patented recombinant endoprotease ss-Kex1-C_611_[10] and the released filgrastim was purified to homogeneity by column chromatography to obtain a clinical grade preparation coded BK0023. Bulk BK0023 in pH 4.5 buffered solution, was sterile filtrated, formulated in glass vials containing for each milliliter 300 μg filgrastim, 50 mg sorbitol, 0.04 mg polysorbate 80, 0.59 g acetate ions 0.83 mg sodium ions and stored at 2-8°C. The reference filgrastim was Neupogen^®^ 300 μg/ml, (Dompé Biotec, Italy) purchased from a pharmacy.

### Sodium dodecyl sulphate polyacrylamide gel electrophoresis (SDS-PAGE) and Western Blotting

SDS-PAGE was carried out in reducing and non-reducing conditions on polyacrylamide gels (4% stacking gel, 12% running gel) prepared according to Laemmli [11]. Samples were diluted 1:1 v/v with 62.5 mM Tris–HCl-pH 6.8-25% glycerol-2% SDS-0.01% Bromophenol Blue ± 5% 2-mercaptoethanol buffer and heated for 5 min at 95°C. Ten microliters samples (3–5 μg of protein) were loaded in the gels which, after the electrophoretic migration, were stained with Coomassie Brilliant Blue R 250 and washed with 10% v/v acetic acid-15% v/v ethanol solution.

Western Blotting was carried out, after SDS-PAGE separation and electrophoretic transfer, using anti-G-CSF rabbit polyclonal antibody (PeproTech, Hamburg, Germany) as primary antibody and anti-rabbit goat antibody HRP conjugate (Santa Cruz Biotechnology, Heidelberg, Germany) as secondary antibody.

### Reversed Phase-High Pressure Liquid Chromatography (RP-HPLC)

BK0023 purity and contamination with its oxidized forms were detected by RP-HPLC of 3–5 μg/10 μl samples performed on a Symmetry C18 300 Å column, 2.1 × 150 mm, 3.5 μm (Waters Corp., Milford Massachusetts, USA) with a HP 1100 liquid chromatography system (Agilent Technologies, Waldbronn, Germany) equipped with automatic sample injection and UV detection at 215 nm. The column was equilibrated in 46.3% buffer A (HPLC grade H_2_O containing 0.1% trifluoroacetic acid) and 53.7% buffer B (acetonitrile containing 0.08% trifluoroacetic acid). Gradient elution was carried out at a flow rate of 0.285 ml/min and 53°C as follows: from 53.7% to 58.3% buffer B in 8 min; from 58.3% to 63,3.6% buffer B in 8 min; from 63.3% to 72.7% buffer B in 6 min.

### Size-Exclusion High-Pressure Liquid Chromatography (SE-HPLC)

BK0023 contamination with its aggregated forms was detected by SE-HPLC of 5 μl samples performed on a TSKgel Super SW2000, 4 μm, 4.6 × 30 cm column (Tosho Bioscience, Stuttgart, Germany) with a HP 1100 liquid chromatography system and UV detection at 214 nm. The column was equilibrated and isocratically eluted with 63 mM phospate-pH 7- 3% isopropanol mobile phase at a flow-rate of 250 μl/min and 25°C.

### Amino acid sequence analysis and peptide mapping

Amino acid sequences of BK0023 and Neupogen^®^ were compared by NH_2_-terminal amino acid sequence analysis of the first 15 residues by standard Edman degradation procedure on an automated Procise 610A Protein Sequencer (Applied Biosystems, Foster City CA, USA) and by peptide mapping after proteolysis in non-reducing and reducing conditions with endoprotease Glu-C from *S aureus* followed by RP-HPLC separation of resulting peptides and MS/MS identification. Mass spectrometry was performed in positive ion mode on a MALDI-TOF Reflex III instrument (Bruker, Bremen, Germany).

### Assay of process derived contaminants

Endotoxin contamination of formulated BK0023 was assayed by Lymulus Amoebocyte Lysate (LAL) test using Pyrochrome LAL kit (Cape Code Inc, Falmouth, MA, USA), while residual *E.coli* proteins were detected by immunoenzymatic method assay using an *E.coli* HCP Elisa kit (Cygnus Technologies. Wrentham, MA, USA). Both tests were performed according to the manufacturers’ instructions.

Quantization of host/vector DNA contamination has been performed under Good Laboratory Practice conditions by an external contractor (NewLab Bioquality AG, Erkrath, Germany).

### Stability study

A three months stability study of formulated BK0023 was performed maintaining aliquots of product solutions at 5 ± 3°C and 25 ± 2°C. At time intervals, samples were assayed by RP- and SE-HPLC to detect the appearance of new minor degradation peaks.

### In vitro cell proliferation

Cell proliferation effect of BK0023 in comparison to Neupogen^®^ was measured on a murine myeloblastic NFS-60 cell line according to a described colorimetric method [12]. Briefly, NFS-60 cells were cultured at a confluence of 5 × 10^5^ cells/ml in RPMI 1640 medium containing 200 mM L-glutamine, 1 mM Na-pyruvate, 10% FBS and 33 IU/ml of mouse IL 3 at 37°C, under a 5% CO2 humidified atmosphere. After a starvation period of 17 hours without serum, 1 × 10^4^ cells/well were seeded in triplicate in microtiter plates and incubated for 48 hours with samples in concentration ranging from 0.0015 to 5 ng/ml. At the end of incubation, 20 μl of WST-1 cell proliferation reagent were added and, after 4 hours of incubation, the formation of soluble formazan dye was estimated by reading the absorbance at 450 nm with an ELISA plate reader. EC_50_ of BK0023 and Neupogen were calculated from the sigmoidal concentration-response curve using the GraphPad Prism program (GraphPad Software, San Diego, CA, USA). Raw data for the parallel line method were processed according to the European Pharmacopoeia [13] and the results were expressed as rate of BK0023 towards Neupogen^®^’s potency.

### Radioligand binding assay

The potencies of BK0023 and Neupogen^®^ in displacing ^125^I-radiolabelled h-G-CSF (Amersham Biosciences, Amersham, UK) bound to the G-CSF receptor were determined using NFS-60 cell line as described [14]. The experiments were carried out in duplicate by incubating 2.5 × 10^6^ cells for 1 hour at 37°C in 150 μl of RPMI 1640 medium containing 10% FCS, 20 mM Hepes-NaOH (pH 7.3), 500 pM [^125I^]G-CSF and increasing concentrations of either BK0023 or Neupogen^®^.

After the incubation, the tubes were chilled on ice and the mixtures were layered over 400 μl of phtalate oil (dibutylphtalate: dioctylphtalate 3:2) in 1.5 ml Eppendorf tubes and centrifuged at 6,000 g for 10 min at 4°C. Following aspiration of the aqueous and organic supernatants, the pellets were solubilized and mixed with Formula-989 liquid scintillation cocktail (Perkin-Elmer, Monza, Italy). The radioactivity was determined by liquid scintillation counting in a TRI-CARB 1600TR liquid scintillation counter (Canberra-Packard, Cassina de’ Pecchi, Italy) with an efficiency of 70%. Nonspecific binding was determined in the presence of 500 nM unlabeled r-G-CSF. Assays were performed in duplicate.

### Immunoenzymatic analysis

BK0023 and Neupogen^®^ concentrations were measured in rat plasma samples by an ELISA method using the commercial kit G-CSF Instant ELISA (Bender Med. System, Vienna, Austria) according to the the manifacture’s instructions.

### Preclinical studies

Care and handling of animals used for preclinical studies were in accordance with the provisions of the European Economic Community Council Directive 86/209 recognized and adopted by the Italian Government with the Ministerial Decree No. 230/95-B and the NIH publication No. 85–23, revised in 1985.

Seven-nine week old Cr:CD Sprague Dawley rats and 13–18 week old New Zealand White female rabbits were purchased from Charles River Srl (Calco, Italy) and stabulated for acclimatization for about 4 and 2 weeks, respectively, under thorough observation by a veterinarian.

### Local tolerability of BK0023 in rabbits

Local tolerability after a single subcutaneous, intravenous (into ear vein) or intramuscular injection of 300 μg/ml/rabbit of formulated BK0023 was studied using 6 female rabbits for each administration route.

The reaction to treatment was assessed by visual inspections on the day of treatment and on the day after in the first 3 animals of each group and for 14 days in the remaining animals*.* For microscopic evaluation, three animals from each group were killed 48 hours after treatment, while the remaining animals from each group were killed 14 days after dosing.

### Repeated dose toxicity in rats

Rats of both sexes (10 males and 10 females for each group) were treated for 4 weeks by daily subcutaneous injection of formulated BK0023 or Neupogen^®^ at dosage of 20, 100 and 500 μg/kg/day. Upon treatment completion, the animals were observed for a further 4 week recovery period.

All the animals were submitted to routine clinical observations (local tolerance, clinical signs, body weight, food and water consumption and ophthalmoscopic examination), standard laboratory analyses (blood chemistry, haematology and urinalysis) and post-mortem examinations (autopsy, organ weight and histology).

The pharmacodynamic parameters (white blood cells and neutrophil counting) were determined by withdrawing blood samples into tubes containing EDTA as anticoagulant before the start of treatment, at days 14 and 28 and upon completion of the recovery period. Blood samples were stored at 4°C and used for counting white blood cells (WBC) and neutrophils (ANC) on a Animal Blood Counter ABC (ABX Diagnostic, Montpellier, France) according to manufacturer’s instructions.

The toxicokinetic parameters were determined by collecting serum samples at 1, 2, 4, 8 and 24 hours post-dose on day 1 and on day 14 from all the treated animals. The serum h-G-CSF concentration was measured using an ELISA technique. The formation of anti-G-CSF antibodies was also evaluated by competitive ELISA assay to examine whether a relationship existed between antibody response and toxicokinetics.

### Pharmacodynamics and pharmacokinetics in neutropenic and non-neutropenic rats

Male Crl:CD rats were allocated in 12 groups of 10 animals, while one group of 6 animals was used as a control. 6 groups of 10 animals were treated by intraperitoneal administration at day -1 with a single 50 mg/kg dose of cyclophosphamide to induce a marked increase of neutropenia, lasting for several days and of comparable severity to WHO grade III neutropenia in humans [15]. All animals were then treated for 4 consecutive days by subcutaneous injections of 10, 30 and 100 μg/kg of BK0023 and Neupogen^®^ while control group was administered the vehicle alone.

The pharmacodynamic parameters (WBC and ANC) were determined on blood samples withdrawn from the tail vein of the first 6 rats taken from each group at the following time points: pre-dose, 1, 2, 3, 4, 5, 6, 8, 10 and 12 days after the first dose.

In addition, pharmacokinetics was investigated by collecting serum samples at 6 time points (pre-dose, 1, 2, 4, 8 and 24 hours after dosing) on day 1 of administration from the remaining 4 animals of each treatment group. The sera were stored frozen pending the determination of G-CSF concentration with a commercial ELISA kit.

### Phase I clinical study

Evaluation of pharmacodynamic equivalence and pharmacokinetics bioequivalence of BK0023 to Neupogen^®^ were assessed by an external Clinical Research Organization in healthy male volunteers after single and multiple-dose subcutaneous administration according to an experimental protocol authorized by Canton Ticino Ethical Committee and by the Swiss Central Health Authority.

Healthy men were treated with formulated BK0023 or Neupogen^®^ by subcutaneous injections at the following doses according to a cross-over design in 2 consecutive study periods, separated by a wash-out period of at least 28 days.

Group 1 (16 subjects): 2.5 μg/kg/day for **7** consecutive days;

Group 2 (16 subjects): 5 μg/kg/day for **7** consecutive days;

Group 3 (16 subjects): 10 μg/kg/day for 5 consecutive days.

The primary clinical phase I study endpoints were as follows:

1) Equivalence in terms of pharmacodynamic effects of BK0023 versus Neupogen^®^ assessed on baseline adjusted increase of ANC.

2) Bioequivalence of pharmacokinetic profile of BK0023 and Neupogen^®^ assessed as peak concentration of G-CSF (Cmax) and area under the curve (AUC_0-24 hours_) for BK0023 and Neupogen at both day 1 and at steady-state (last treatment day).

The secondary clinical phase I study endpoints were as follows:

1) Equivalence in terms of induction of CD34^+^ cells of BK0023 versus Neupogen^®^ assessed on CD34+ peak concentration and AUC_CD34+_ versus time from day 1 to 10 (2.5 and 5 μg/kg/day dose groups) or from day 1 to 8 (10 μg/kg/day dose group)

2) Bioequivalence in terms of G-CSF peak concentration of BK0023 and Neupogen^®^ at both day 1 and at steady-state (last treatment day)

3) Bioequivalence of pharmacokinetic parameters of BK0023 and Neupogen^®^ at both day 1 and at steady-state (last treatment day) assessed as T_max_ (time to peak concentration), t_1/2_ (half life) and clearance.

4) Safety and tolerability of BK0023 as compared to Neupogen^®^

The study was designed as a group sequential clinical trial in order to recalculate the correct sample size necessary to obtain statistically acceptable pharmacodynamic and pharmacokinetic results for each group, based on the intra subject variability, to achieve the primary study objectives.

Consequently, the study was conducted in two parts:

Part 1: 16 subjects per dose group were enrolled and treated with both drug products in cross-over. The pharmacodynamic and pharmacokinetic primary parameters, that is the AUC of ANC, the maximal attained ANC and the serum filgrastim AUC on day 1 and at steady state were calculated after the end of the second period of each dose group. A pharmacokineticist, independent from the clinical study management and from the sponsor*,* temporarily broke the blind with the aim of evaluating intra-subject variability. The biostatistician re-calculated the sample size with the actual study *ad interim* results in order to verify whether additional subjects were needed to obtain statistically valid results.

Part 2: On the basis of sample size re-calculation, an additional number of subjects for each dose group was enrolled: 16 subjects in the Group 1 (2.5 μg/kg/day), 20 subjects in the Group 2 (5 μg/kg/day) and 6 subjects in the Group 3 (10 μg/kg/day).

The pharmacodynamic effects of both BK0023 and Neupogen^®^ were determined by ANC performed on whole blood samples with a fully automated laser ADVIA 120 Haematology System (Bayer) which incorporates flow cytometric principles and cytochemistry and by evaluating the count of peripheral blood CD34 positive (CD34^+^) hematopoietic stem cells by flow cytometric analysis as described [16]. In fact, beside regulating granulopoiesis, G-CSF is also able to mobilize CD34^+^ cells from the marrow to the blood enabling the use of stem cell enriched peripheral blood in hematopoietic transplantation in lieu of bone marrow cells [17].

The pharmacokinetic profile of filgrastim was determined in serum samples withdrawn after the 1st and the last dose and stored frozen until the determination of G-CSF concentration with a commercial ELISA kit

### Statistical analysis of clinical and preclinical data

Results were analyzed using SAS^®^ version 9.1.2 for Windows software. The AUC of ANC and CD34^+^ cells and their maximal attained counts were compared based on analysis of variance for a cross-over design: the 95% CI for the ratio of the averages (population geometric means, if parameters are log-transformed) of the values for the test and reference and the two onesided t-tests of Schuirmann at the level of significance of 2.5%. Criteria for equivalence on ANC parameters were that the 95% CI of the ratio between treatment means fell within 85%-115% (or 85%-118% when parameters are log-transformed). AUC and peak concentration of serum filgrastim were compared based on Latin-square analysis of variance for a cross-over design: the 90% CI for the ratio of the averages (population geometric means) of the values for the test and reference and the two one-sided t-tests of Schuirmann at the level of significance of 5%. Criteria for bioequivalence were that the 90% CI of the treatment means ratio fell within the range of 80 to 125%. Pharmacokinetic data were calculated using Kinetica™ Version 4.4.1 software (Thermo Electron Corporation, USA); the area under the blood concentration-time curve (AUC) was calculated using the trapezoidal rule.

Experimental data of in vitro inhibition of radioactive G-CSF binding by BK0023 and Neupogen to cell expressing G-CSF receptor were analyzed by using the GraphPad Prism Program (San Diego, USA).

Information was given in both oral and written form to each volunteer. Before being admitted to the clinical study subjects expressed their consent to participate singing copy of the written informed consent form.

## Results

### Analysis of physico-chemical properties

Purified BK0023 was characterized by a panel of chemico-physical methods, including amino acid sequence analysis of NH2-terminal residues, RP-HPLC/MS mapping of proteolytic products, MALDI-TOF-MS, SDS-PAGE and Western blotting. The results showed the absolute comparability to the clinically used filgrastim commercialized under the brand name of Neupogen^®^ as well as levels of process- and product-related contaminations within the limits accepted by regulatory authorities (Tables 1, 2 and 3).

**Table 1:** Analytical comparison between BK0023 and Neupogen^®^

**Parameter**	**Analysis**	**Results and notes**
**Structural and conformational characterisation**	First 15 N-terminal residues by automatic Edman degradation	Results agree with the expected sequence
	Reducing and non-reducing Glu-C proteolysis and RP-HPLC/MS mapping	Primary structure and disulfide bridge positions according to the expected sequence
	MALDI-TOF-MS	According to the calculated value
	SDS-PAGE	Migration profile comparable to Neupogen^®^
	SE-HPLC	Elution profile comparable to Neupogen^®^
**Identity**	Western blotting	Binding to specific antibody as Neupogen^®^
	RP-HPLC	Elution profile comparable to Neupogen^®^

**Table 2:** Product related impurities of purified bulk BK0023 preparations

**Impurity**	**Method**	**Result**
BK0023 oxydized forms	RP-HPLC	< 1%
BK0023 aggregate forms	SE-HPLC	< 1%

**Table 3:** Process related impurities of formulated BK0023 preparations

**Impurity**	**Method**	**Result**
Bacterial endotoxins	LAL test	< 5 EU/mg
Host proteins (ECP)	Immunoenzymatic assay	< 20 ppm
Host/vector DNA	Hybridation to degenerated probe	< 100 pg/mg

*EU* Endotoxin unit, *ppm* part per million parts, *pg* picograms.

The result of the stability study of formulated BK0023 are reported in Table 4. While the drug product appeared stable at +5°C over a three month period, protein degradation and aggregation occurred to a limited extent when the product was stored at 25°C. Consequently, all BK0023 preparations were stored refrigerated as also recommended for Neupogen^®^.

**Table 4:** Stability of formulated BK0023 maintained at 5 and 25°C. Aliquots of samples were assayed at time 0, 1 and 2 months by RP-HPLC and SE-HPLC

**Assay**	**Storage time**	**Temperature 5°C**			**Temperature 25°C**		
		BK0023 mg/ml	Main peak%	Sum of impurities%	BK0023 mg/ml	Main peak%	Sum of impurities%
**RP-HPLC**	0	0.29	99.6	0.4	0.29	99.6	0.4
	1 month	0.29	99.6	0.4	0.28	99.6	0.3
	3 months	0.28	99.6	0.4	0.28	99.1	0.9
**SE-HPLC**	0	0.28	99.6	0.4	0.29	99.6	0.4
	1 month	0.27	99.6	0.4	0.28	99.6	0.3
	3 months	0.28	99.6	0.4	0.28	99.1	0.9

Aliquots of samples were assayed at time 0, 1 and 2 months by RP-HPLC and SE-HPLC. Peak purity and total impurities (or aggregates in the case of SE-HPLC analysis) are reported as percentage of total peak areas.

### In vitro studies

Two studies, namely a cell proliferation assay and a radioligand binding assay using the murine myeloblastic NFS-60 cell-line were carried out to compare the *in vitro* biological activity of BK0023 and Neupogen^®^.

The results of proliferation assay indicated that BK0023 and Neupogen displayed *in vitro* the same biological potency as demonstrated by very close EC_50_ values (respectively 43.90 ng/ml ± 1.16 S.E.M. and 38.59 ng/ml ± 1.24 S.E.M.) overlapping at 95% confidence intervals (with respective range of 31.97-60.29 and 24.61-60.41 ng/ml).

**Figure 1:**
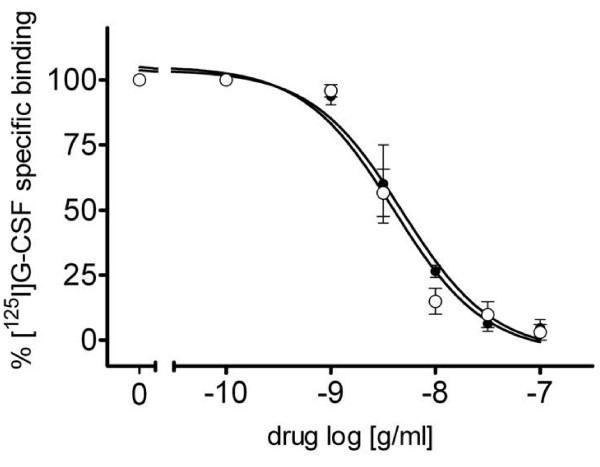
**Concentration-dependent inhibition of [**^**125**^**I]-G-CSF binding by BK0023 (black circle) and Neupogen^®^ (open circle) in NFS-60 cells expressing G-CSF receptor.** Data are reported as percent of specific binding at each concentration of displacer and are the means ± S.E.M. of four separate experiments. Statistical analysis by Student’s t-test did not show any significant difference between BK0023 and Neupogen^®^.

The radioreceptor assay also showed the same competition for BK0023 and Neupogen^®^ towards radiolabelled r-h-G-CSF bound to G-CSF receptors in NFS-60 cells with estimated IC_50_ respectively of 4.52 ± 0.65 and 5.89 ± 0.49 ng/ml with no statistically significant difference (Figure 1).

### Local tolerability studies

Local tolerability was assessed in rabbits by a single intravenous, subcutaneous and intramuscular administration of 300 μg of BK0023, which is the highest concentration given in humans as a bolus injection. Slight erythemas or oedemas, which were rapidly and completely reversible, were seen at sites of intravenous and subcutaneous injections while no significant reaction was observed in animals treated by intramuscular route. In any case, no abnormality was detectable by microscopy examination as well as no clinical abnormal signs were seen in any animal. These results indicate that BK0023 is well tolerated locally.

### Toxicity studies in rats

A repeated-dose toxicity study was performed in rats to compare the toxicological profile of BK0023 and Neupogen^®^ administered subcutaneously for 4 weeks at dosages of 20, 100 and 500 μg/kg/day, corresponding to 2–4, 10–20 and 50–100 times the clinical dose in humans (5–10 μg/kg/day), respectively. In the same study we compared the pharmacodynamic and the immunotoxicologic profiles of the two products after 28 days of daily subcutaneous administration. The study also included a comparison of the toxicokinetic profiles after 14 days of treatment and an assessment following a 4-week recovery period.

The results showed that administration of BK0023 and Neupogen^®^ produced a comparable significant dose-related increase in neutrophils and white blood cells with respect to the control group (data not shown) indicating that the animals received systemic exposure to both products in a dose-related manner.

As regards the blood chemistry analyses, serum alkaline phosphatase concentration significantly increased in all the treated animals according to a dose-related trend (Table 5). This effect may be correlated to osteoblast and osteoclast cell function which, in turn, is indicative of BK0023 and Neupogen^®^ activity [18] .

**Table 5:** Alkaline phosphatase (U/liter) assayed in blood of both female and male rats treated subcutaneously for 28 days with 20, 100 or 500 μg/kg/day with BK0023 or Neupogen^®^

**Dose**	**Control**		**BK0023**		**Neupogen^®^**	
	**Males**	**Females**	**Males**	**Females**	**Males**	**Females**
**0 μg/kg/day**	440 ± 110	264 ± 69				
**20 μg/kg/day**	-	-	669* ± 130	429 ± 135	773** ± 90	400** ± 50
**100 μg/kg/day**	-	-	1365** ± 303	747** ± 212	1022** ± 127	855** ± 166
**500 μg/kg/day**	-	-	2109.** ± 538	1227** ± 268	2378.** ± 647	1005** ± 367

Data are reported as mean values ± SD.

* = mean value of group is significantly different from the control group at P < 0.05.

** = mean value of group is significantly different from the control group at P < 0.01.

Statistical analysis was performed according to Dunnett’s test.

Statistically significant increases in urea and creatinine levels in females and decreases in chloride levels in males were also observed in animals receiving the highest dose of Neupogen^®^, while other statistically significant changes were sporadically recorded in animals receiving the lower doses of BK0023 and Neupogen^®^. However, these differences were considered too small to be toxicologically meaningful and were therefore considered to have no toxicological implication.

No difference in body weight or food consumption and no ophthalmic lesions were observed during the study. The only anomalous *in vivo* observation was a swelling of the lower hind limbs in both BK0023 and Neupogen treated rats, which is an already described G-CSF treatment related effect possibly due to induction of fluid retention [19]. Of note, all changes observed during treatment were no longer detected following the 4-week recovery period, thereby confirming complete reversibility. The immunogenic response to BK0023 was comparable to that of Neupogen^®^ for anti-G-CSF immunoglobulins were detected in 3 serum samples out of 120 (1 male and 1 female rat treated with 20 and 500 μg/kg/day of BK0023 and 1 female rat treated with 500 μg/kg/day of Neupogen^®^) at the end of recovery period. Moreover, in a separate study in rats, no specific antibodies against r-h-G-CSF were detected following daily administration of BK0023 at a concentration of 200 μg/kg/day over a 4-week period. The toxicokinetics of BK0023 and Neupogen^®^ assessed by comparing the pharmacodynamic parameters after 1, 14 and 28 days of treatment as well as at the end of 4-week recovery period did not show any significant difference (data not shown).

### Pharmacodynamic and pharmacokinetic studies in normal and neutropenic rats

Since G-CSF stimulates the production of bone marrow cells interacting with a specific surface receptor, the increase of ANC is a direct biochemical index of efficacy. Thus, white blood cell count and ANC were determined during 4 consecutive days of subcutaneous administrations to normal and cyclophosphamide treated neutropenic rats at the doses of 10, 30 and 100 μg/kg of BK0023 and Neupogen^®^. The dose range was selected to induce a marked dose–response effect in terms of degree and duration of neutrophil production stimulation in order to detect any difference between BK0023 and Neupogen^®^.

Pharmacodynamic evaluations demonstrated that both BK0023 and Neupogen^®^ boosted the proliferation of white blood cells (WBC) and in particular of neutrophil cells, which appeared hypersegmented, indicating complete activation.

**Figure 2:**
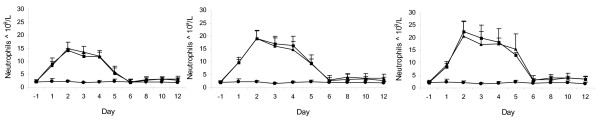
**Mean neutrophile counts (× 10**^**9**^**/l) obtained in groups of 10 normal rats treated for 4 days by subcutaneous injections of 10, 30 and 100 μg/kg/day of BK0023 and Neupogen^®^ are respectively shown in left, central and right panels where full circles, squares and triangles are respectively referred to control rat, BK0023 and Neupogen.** No significant difference was found between BK0023 and Neupogen treated groups analysed by Student’s test. Significant differences between treated and control groups analyzed by Dunnet’s test were found at P < 0.05 (*) or P < 0.01 (**).

**Figure 3:**
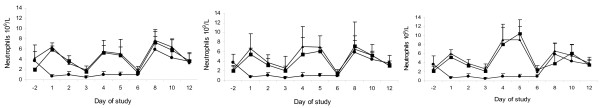
**Mean neutrophile counts (× 10**^**9**^**/l) ± SD obtained in groups of 10 neutropenic rats treated for 4 days by subcutaneous injections of 10, 30 and 100 μg/kg/day of BK0023 and Neupogen^®^ are respectively shown in left, central and right panels where full circles, squares and triangles are respectively referred to control rats, BK0023 and Neupogen.** No significant difference was found between BK0023 and Neupogen treated groups analysed by Student’s test. Significant differences between treated and control groups analysed by Dunnet’s test were found at P < 0.05 (*) or P < 0.01 (**).

While both products yielded a comparable and dose–related increase in total WBC and ANC, no difference was observed regarding the other blood cells (lymphocytes, eosinophils, basophils, monocytes and large unstained cells) in either the neutropenic or normal rats (data not shown). In normal rats, neutrophil levels increased during the first day of treatment and returned to normal values approximately 2 days after the last treatment (Figure 2). Differently from normal rats, neutropenic rats exhibited a consecutive cycle of increasing and decreasing of the neutrophil level in response to either BK0023 or Neupogen^®^ (Figure 3). The first increase may be due to the mobilisation of the residual pool of neutrophils after cyclophosphamide administration, while the subsequent decrease may be related to the cyclophosphamide-dependent reduction of neutrophil production by the bone marrow blasts. The second increase could depend on the effect of BK0023 and Neupogen^®^ leading to an increase of cells from the myeloid series.

The results of the pharmacokinetics study on day 1, summarized in Table 6, showed that filgrastim was systemically absorbed in both non-neutropenic and neutropenic rats after both BK0023 and Neupogen^®^ and that the animals were similarly exposed to drugs; some sporadic differences between treatments, shown in Table 6, can be ascribable to the intrinsic animal variability or to different drug absorption after subcutaneous administration.

**Table 6:** Major pharmacokinetic parameters for BK0023 and Neupogen^®^ administered to normal and cyclophosphamide neutropenic rats following a single subcutaneous administration of 10, 30 and 100 μg/kg

		**Normal rats**			
**Product**	**Nominal dose μg/kg**	**Cmax ng/ml**	**Tmax hour**	**AUC ng · h/ml**	**t½ hour**
**BK0023**	10	51	2	179	1.5
	30	64	2	377	2.6
	100	377	2	1582	2.6
**Neupogen^®^**	10	14	1	65	1.8
	30	68	2	369	2.4
	100	272	1	1121	2.2
		**Neutropenic rats**			
**BK0023**	10	27	2	134	1.9
	30	84	2	410	1.7
	100	444	2	1558	1.8
**Neupogen^®^**	10	31	2	132	2.0
	30	87	2	485	2.5
	100	309	2	1620	2.4

Abbreviations: *AUC* Area under the serum concentration-time curve from time zero to infinity, *C*_*max*_ Maximum observed serum concentration, *T*_*max*_ time to C_max,_*t*_*(1/2)*_ Elimination half-life associated with terminal shape (λ_2_) of a semilogarithmic concentration-time curve.

Summarizing, preclinical *in vivo* studies showed that BK0023 and Neupogen^®^ increased the proliferation of neutrophil cells in normal rats in a similar dose-dependent manner while in neutropenic rats, both products were capable of controlling myelotoxicity with the same dose-dependent recovery of WBC and neutrophil counts. In addition, pharmacokinetic parameters indicated that filgastim was well absorbed in non-neutropenic and neutropenic rats and cleared from blood according to a comparable pharmacokinetic profile after both BK0023 and Neupogen^®^.

### Phase I clinical study

**Figure 4:**
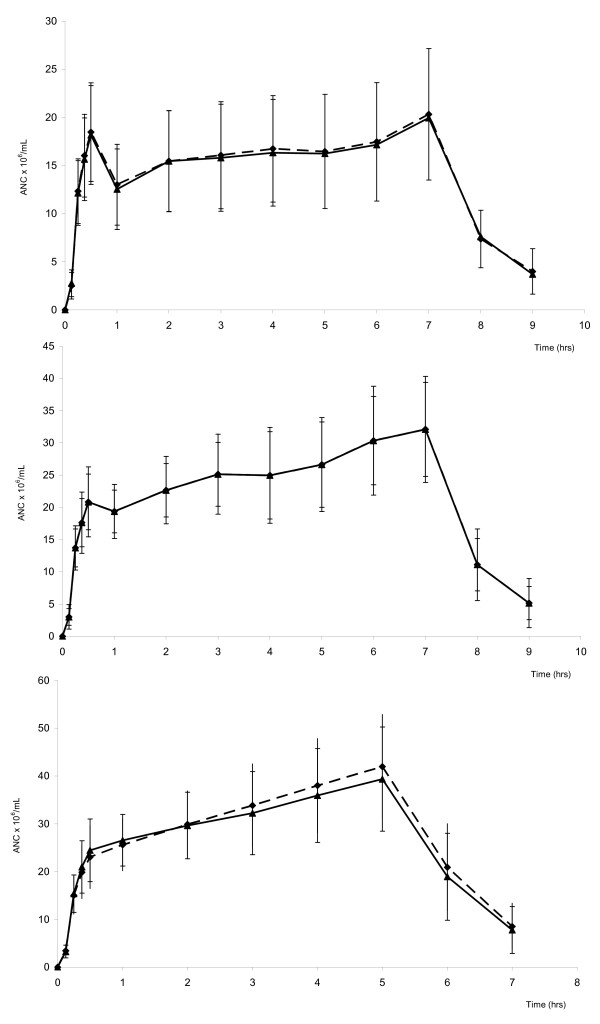
**Mean neutrophil counts (× 10**^**3**^**/μl) ± SD measured in human healthy volonteers treated for 7 days by subcutaneous injections of BK0023 (triangles) and Neupogen^®^ (circles) at dosage of 2.5 μg/kg/day (upper panel) and 5.0 μg/kg/day (central panel) and for 5 days by subcutaneous injections of BK0023 and Neupogen^®^ at dosage of 10.0 μg/kg/day (lower panel).**

A phase I clinical study was performed in healthy male volunteers treated with BK0023 and Neupogen^®^ by subcutaneous injection of 2.5 and 5 μg/kg/day for 7 consecutive days and of 10 μg/kg/day for 5 consecutive days in order to assess the equivalence between the two products in terms of pharmacodynamic effect and the bioequivalence in terms of pharmacokinetic profile. The count of neutrophils and CD34^+^ cells were quite similar in both the rate and extent after a single injection of the two products on day 1 and after a multiple injection treatment of 5 days as shown, for example, in Figure 4 which refers to ANC. Concerning the CD34+ cell concentration, the baseline corrected values measured in 22 subjects up to day 8 after a 5-day treatment with daily injection of 10 μg/kg of BK0023 or Neupogen were respectively of 93,37 ± 35,33 and 95,40 ± 42,59 (mean CD34^+^_max_/μL ± SD) and of 8166 ± 2917 and 8670 ± 3797 (mean AUC_CD34+_/μL x h ± SD). Data analysis performed for each dose group showed no statistically difference for ANC and CD34^+^ cell count as well as for areas under the curve of both cell types (data not shown). In other words, BK0023 was equivalent to Neupogen^®^ in terms of both AUC and maximal attained level of ANC and CD34^+^ cells.

**Figure 5:**
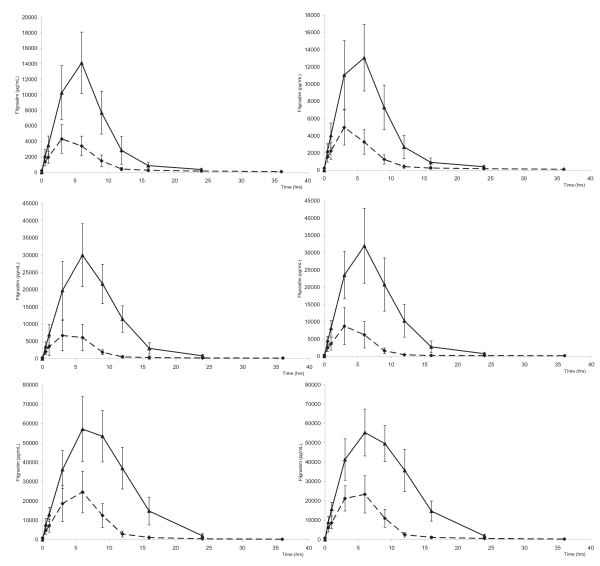
Plasma level profiles of G-CSF (pg/ml) measured by immunoenzymatic assay at day 1 (solid lines) and at the last day of treatment (dashed lines) for BK0023 2.5 μg/kg/day (A); Neupogen 2.5 μg/kg/day (B); BK0023 5 μg/kg/day (C); Neupogen 5 μg/kg/day (D); BK0023 10 μg/kg/day (E); Neupogen 10 μg/kg/day (F).

Time-dependent serum concentration profile of filgrastim measured by immunoenzymatic assay at day 1 and at last day of subcutaneous treatment with BK0023 and Neupogen at dose levels of 2.5, 5 and 10 μg/kg/day are reported in Figure 5 which shows that pharmacokinetic profiles of serum filgrastim were superimposable both at day 1 (after a single administration) and at steady-state conditions (after 7 daily injections of 2.5 and 5.0 μg/kg/day or 5 daily injections of 10.0 μg/kg/day).

The corresponding calculated pharmacokinetic parameters Cmax and AUC_0-24 hours_ for BK0023 and Neupogen at both day 1 and at steady-state (last treatment day) showed that parameters of BK0023 treated subjects were similar to those of the Neupogen treated ones (Table 7). For both products, a significant reduction of serum levels of G-CSF (Cmax) and AUC was observed at the last day of treatment versus that obtained at day 1. This effect, which was already described for filgrastim, is due to the enhanced G-CSF clearance through receptor binding, internalization and degradation by the increased neutrophil population [20].

**Table 7:** Pharmacokinetic parameters (mean ± SD) calculated at day 1 and after the last day of treatment (steady-state) for human healthy volonteers treated for 7 days with subcutaneous injections of 2.5 and 5.0 μg/kg/day of BK0023 and Neupogen^®^ and for 5 days with subcutaneous injections of 10.0 μg/kg/day of BK0023 and Neupogen^®^

**Product and dosages**	**First treatment**		**Steady-state**	
	**Cmax ng/ml**	**AUC**_**0-24 hours**_**ng/ml · hour**	**Cmax ng/ml**	**AUC**_**0-24 hours**_**ng/ml · hour**
**BK0023 2.5 μg/kg/day**	14.1 ± 1.4	113.6 ± 30.8	4.7 ± 1.6	32.8 ± 8.5
**Neupogen 2.5 μg/kg/day**	13.4 ± 3.9	111.9 ± 30.3	5.1 ± 2.0	33.7 · 9.6
**BK0023 5 μg/kg/day**	30.1 ± 9.1	276.7 ± 71.6	7.9 ± 4.4	51.2 ± 23.7
**Neupogen 5 μg/kg/day**	32.2 ± 10.7	295.0 ± 86.8	9.0 ± 5.2	55.3 ± 27.0
**BK0023 10 μg/kg/day**	60.1 ± 16.7	670.0 ± 154.9	25.1 ± 10.3	189.4 ± 69.7
**Neupogen 10 μg/kg/day**	56.5 ± 11.8	671.5 ± 122.5	25.1 ± 9.0	188.4 ± 52.2

Statistical evaluation of the pharmacokinetic bioequivalence was also demonstrated by verifying that the 90% confidence interval for the ratio of the means of AUCday1, AUCSS (steady-state AUC), Cmaxday1 and CmaxSS (steady-state Cmax) between treatments met the acceptance interval 80-125% and that the two one-sided t-tests of Schuirmann did not detect any difference at the level of significance of 5% [21].

Pharmacokinetics of filgrastim following treatment with BK0023 and Neupogen^®^ was proved not to differ significantly also when other parameters (like elimination half-life t½, time of peak concentration Tmax and systemic clearance Cl/body weight) were compared at day 1 of treatment and at last day of subcutaneous treatment by the non-parametric Kruskal-Wallis test [22].

In the whole clinical study, adverse effects were reported for 70/102 subjects after treatment with BK0023, as compared with 67/102 subjects after treatment with Neupogen^®^ for a total of 235 and 261 treatment-related adverse effects for BK0023 and Neupogen treated subjects, respectively.

The most frequent adverse effect was back pain of mild to moderate intensity which reached a frequency of 65% of subjects with BK0023 at the dose of 5 μg/kg/day. followed by bone pain which reached a frequency of 47% of subjects after treatment with Neupogen^®^ at the dose of 10 μg/kg/day. Headache frequency between 8 and 16% of patients treated with at 2.5 to 10 μg/kg/day of BK0023 and Neupogen was also observed. When compared by χ^2^ test, no significant statistical difference was detected in either the number of subjects experimenting any adverse affect and the total number of treatment-related adverse effects reported during the study. All observed treatment-related adverse effects were expected since they are linked to the mechanism of action of G-CSF and were already described for filgrastim [23,24]. It is worth noting that all observed adverse effects were transient, resolved spontaneously and did not lead to discontinuation or treatment withdrawal.

## Discussion and conclusion

We report the preclinical and phase I clinical comparison of an intended biosimilar filgrastim or r-Met-G-CSF (coded BK0023), produced according to an original recombinant process, with the brand filgrastim product Neupogen^®^, which is largely used for decreasing the duration of febrile neutropenia in oncological patients treated with myelosuppressive anticancer drugs. Due to the same analytical and structural profile, BK0023 is expected to display the same tolerability and toxicity profile of Neupogen^®^ as well as the same efficacy as Neupogen^®^ in controlling myelotoxicity induced by chemotherapy during treatment of solid and haematological tumors

The results reported in this paper confirm that BK0023 and Neupogen^®^ display similar *in vitro* biological potency in terms of both stimulation of proliferation of the G-CSF dependent NFS-60 cell line and competition with radiolabeled r-h-G-CSF for binding to G-CSF receptor in NFS-60 cells. BK0023, when locally administered to rabbits at the maximal concentration expected to be used clinically as a bolus injection, is well tolerated without onset of systemic toxicity after single intravenous, subcutaneous or intramuscular injection. In this study, the subcutaneous and intravenous administration routes have been chosen since these are the most common administration routes of filgrastim in humans while the intramuscular route has been included in consideration of accidental product exposure.

BK0023 and Neupogen^®^ were also compared in a subchronic toxicity study by subcutaneous administration in rats, which are a relevant animal model for toxicological studies since r-h-Met-G-CSF is pharmacologically active in this species. In fact, data from literature show that r-h-Met-G-CSF not only increases neutrophil counts in peripheral blood but also enhances neutrophil functions in rats [15].

The results of repeated dose toxicity in rats treated for 28 days with 20, 100 and 500 μg/kg/day (which correspond respectively to 2–4, 10–20 and 50–100 times the clinical dose of 5–10 μg/kg/day) showed for the two products comparable toxicokinetic and immunotoxicologic profiles. Besides, no treatment-related effects were observed at the post-treatment recovery period of 4 weeks so that the maximal administered dose of 500 μg/kg/day may be considered as No Observed Adverse Effect Level (NOAEL) for both BK0023 and reference Neupogen^®^. Altogether, the results obtained in this study indicate the pharma-toxicological comparability of BK0023 and Neupogen^®^.

The *in vivo* studies on neutropenic and non-neutropenic rats showed that both BK0023 and Neupogen^®^ boosted the proliferation of neutrophil cells in normal rats in a dose-dependent manner while in neutropenic rats both products are capable of controlling myelotoxicity with dose-dependent recovery of neutrophils and white blood cells. In preclinical studies, no significant difference between the two products was observed. Our data clearly demonstrate that the biological activity in animal models of BK0023 is comparable to that of Neupogen^®^.

Phase I clinical trial in healthy subjects was carried out according to a multiple-dose, randomised, two-period, cross-over design since there are 2 types of pharmacokinetic non-linearity (non-proportional increase with dose and time dependent non-linearity). Dose levels of 2.5 and 5 μg/kg/day were chosen for the pharmacodynamic study since data in the literature show a clear dose–response relationship in terms of the pharmacodynamic parameters over this range [23] while the dose of 10 μg/kg/day was chosen to ensure that the investigated dose range was relevant to the clinical indications and because it is usual in the praxis for filgrastim. The subcutaneous administration was chosen for the pharmacokinetic and pharmacodynamic bioequivalence study since this one is the administration route most commonly used in the clinical setting.

Pharmacodynamic, pharmacokinetic and safety of BK0023 and Neupogen^®^ were compared according to a cross-over design in 2 consecutive periods with a washout of at least 28 days between the last injection of period I and the first of period II.

The results showed that BK0023 has the same pharmacokinetic profile, efficacy and safety as the reference commercial filgrastim Neupogen^®^ and therefore could be further developed to become a convenient option to treat neutropenia in oncological patients.

Finally, the demonstration of bioequivalence between BK0023 and commercial filgrastim enabled the preparation of a new long lasting site-specific monopegylated filgrastim derivative as described elsewhere [25].

## Competing interests

Bioker srl, is a biotech company located in the Scientific and Technological Park of Sardinia (Italy) fully owned by Multimedica Holding. GS is a IRCCS Multimedica employee; GT is CEO of Bioker srl while DC, RS and GO are Bioker’s employees. ADS is employed at Cross Research SA, a CRO which performed the clinical study. Part of preclinical study were performed by GSJ (University of Parma) and PO and SD (University of Cagliari).

GT and GO are coinventors of the granted patents EP1334183B1 and US7,241,609B reported as references in the manuscript.

## Authors’ contributions

D.C. was responsible of study development and manuscript writing. G.T. was responsible of the research project. R.S. and G.O. were involved in experimental design and studies supporting. P.O. and S.D. were involved in preclinical experiments and results elaboration. G.S. and G.S.J. were involved in the development of the clinical study and drafting of the manuscript. A.D.S. was responsible of clinical development. All authors were involved in experiment planning, results interpretation and discussion and final manuscript revision. All authors read and approved the final manuscript.

## Pre-publication history

The pre-publication history for this paper can be accessed here:

http://www.biomedcentral.com/2050-6511/15/7/prepub
